# Effect of nitrogen application rate on soil fungi community structure in a rice-fish mutualistic system

**DOI:** 10.1038/s41598-019-52602-x

**Published:** 2019-11-07

**Authors:** Shumei Cai, Weiguang Lv, Haitao Zhu, Deshan Zhang, Zishi Fu, Hanlin Zhang, Sixin Xu

**Affiliations:** Institute of Eco-Environment and Plant Protection, Shanghai Academy of Agricultural Sciences, 1000 Jinqi Road, Shanghai, 201403 P.R. China

**Keywords:** Microbiology, Environmental impact

## Abstract

Rice-fish mutualistic production systems rationalise the use of water and soil resources in an improved approach to sustainable food production. However, drivers of fungi community structure in paddy soil, including effects of nitrogen (N) application rate, are unclear in these systems. Here, we assessed soil fungi community and soil physicochemical responses in paddy soil to contrasting rates of N application in a rice-fish system. To clarify the mutualistic effects, the rice-fish system was compared with a standard rice monoculture under a 325.5 kg ha^−1^ N application rate. The results showed that N application rate affected abundance of paddy soil fungi (*P* < 0.05). Alpha diversity and richness of fungi were lower in the rice-fish system, but evenness increased with a decrease in N application rate, while the rate of N determined diversity of soil fungi in the rice-fish system. Dominant genera in the two systems differed, and soil physicochemical properties were more important drivers of soil fungi community structure in the rice-fish mutualistic system than in rice monoculture. Total N, available N and P regulated the abundance of dominant fungi. Our results indicate that management of soil fungi may contribute to sustainable agricultural production.

## Introduction

Rice-fish production systems involving the combination of intensive crop production with large-scale aquaculture are an approved method for sustainable agriculture. These systems reduce the use of chemical pesticides and fertilisers, resulting in fewer plant diseases and insect pests, together with improved soil quality, and thus increase rice yield and fish production^1–5^. Studies have shown that rice-fish mutualistic systems can change soil structural properties, including aggregate composition and bulk density^6–8^. In addition, nutrient and matter cycling can be altered in combined systems. For example, organic matter, aeration, and available nitrogen (N) and phosphorus (P) may be increased, and the nutrient content of aquaculture water can be high due to excessive fertilisation and irrigation, non-point source pollution, but greenhouse gas emissions can be decreased^9,10^. Oehme *et al*.^11^ reported that rice-fish systems promote N uptake in rice and N content in rice straw by improving N utilization and increasing soil fertility due to N accumulation. Datta *et al*.^12^ noted that nitrous oxide emissions were lower from a rice-fish system than from a rice monoculture. However, although nitrous oxide release was reduced in the system, methane emissions were increased, thereby leading to an average increase of 82.56% for carbon (C) credit compliance. Feng *et al*.^3^ found that total N and P, available P, ammonium N and nitrate N were lower in a rice-fish system than in a traditional aquaculture system, with production net profit increased by 114.48%. In a more complex rice-crab-fish system, methane emissions were reduced by 22−54% with a high water depth and active soil respiration^13^.

In general, studies of rice-fish systems have focused on soil physicochemical properties, crop production efficiency and greenhouse gas emissions, while minimal attention has been given to the role of microbes in biochemical processes. Soil microbes, especially fungi, play key roles in nutrient transformation, pollutant degradation and crop disease prevention. Therefore, maintaining fungal species diversity is critical for the delivery of sustainable agricultural production systems. While a range of factors may affect fungal diversity, crop planting pattern and nutrient management are likely to be the most important drivers due to their impacts on productivity and nutrient levels^3,14,15^. Long-term N application may affect the activity and community structure of soil microbes, and the ratio of bacteria to fungi, by altering soil physical and chemical properties, such as soil pH and available N content^6,16^. However, to date there have been no reports on how the soil fungal community is affected in rice-fish mutualistic systems. It is therefore important to track how rice-fish mutualistic production systems and nitrogen application rate influence changes in soil fungal community migration.

We hypothesised that the long-term rice-fish symbiosis method may significantly reduce the impact of N input on soil fungus diversity, altering dominant soil fungus species. The objectives of the present study were to assess the community responses of soil fungi in a rice-fish mutualistic production system to different levels of N application. We performed qPCR and Hiseq sequencing to estimate the effects of the rice-fish mutualistic system and N application rate on fungal abundance and community diversity to better understand the role of fungi in soil fertility and sustainable agricultural production.

## Results

### Abundance and diversity of soil fungi

We used qPCR analysis of ITS1 rRNA gene copy number to estimate abundance and diversity of soil fungi. The results showed that the abundance of fungi decreased with a reduction in N application rate in the rice-fish system. Under a 325.5 kg ha^−1^ N application rate, the abundance was lower in the rice-fish system (RSN100) than the rice monoculture (RMN100) (Table 1). Diversity and evenness of fungi measured by the Shannon and Simpson’s indices, respectively, revealed no treatment effects, however in the rice-fish system, species richness (Chao) of treatments RSN90 and RSN50 were significantly lower than in other N application treatments (Table 1).

**Table 1:** Treatment effect on abundance and diversity of soil fungi based on ITS1 rRNA gene sequence assignment dataset, with a 97% sequence similarity threshold.

	ITS1 copies g soil^−1^ (×10^6^)	OTU number	Chao estimated richness	Shannon Diversity Index	Simpson Evenness Index
RMN100	1.51 ± 0.49a	361.33 ± 17.94a	395.00 ± 14.74a	4.26 ± 0.23a	0.04 ± 0.02a
RSN100	1.17 ± 0.52ab	295.67 ± 25.54ab	327.67 ± 30.96ab	3.84 ± 0.47a	0.07 ± 0.03a
RSN90	0.61 ± 0.07ab	282.67 ± 26.69ab	310.67 ± 21.23b	4.21 ± 0.11a	0.04 ± 0.00a
RSN70	0.96 ± 0.26ab	305.33 ± 22.02ab	345.33 ± 20.79ab	4.39 ± 0.09a	0.04 ± 0.01a
RSN50	0.57 ± 0.15b	250.33 ± 25.65b	311.33 ± 18.49b	4.00 ± 0.19a	0.05 ± 0.01a
RSN0	0.79 ± 0.35ab	319.33 ± 28.29ab	363.33 ± 17.05ab	4.10 ± 0.13a	0.05 ± 0.01a

**Figure 1:**
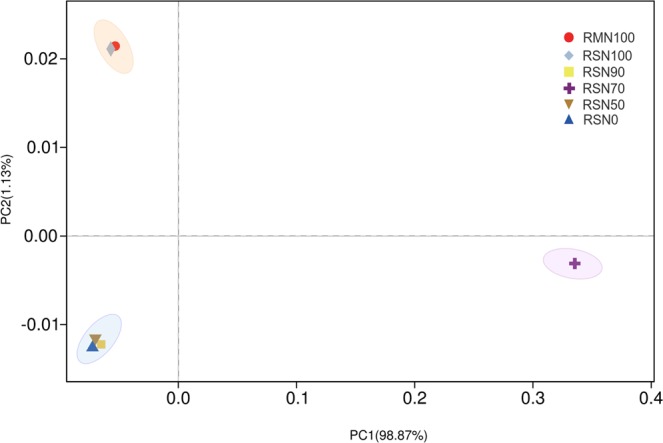
Principal coordinates analysis plot of unweighted-unifrac distances based on genus at 97% similarity.

Principal component analysis (PCoA) of fungi composition at the genus level showed that PC1 accounted for 98.87% of the variation among treatments (Fig. 1). RMN100 and RSN100 treatments clustered together, while RSN0, RSN50 and RSN90 treatments formed another distinct cluster separated by PC2. However, RSN70 was segregated into a unique community driven by PC1.

### Soil fungi community composition

**Figure 2:**
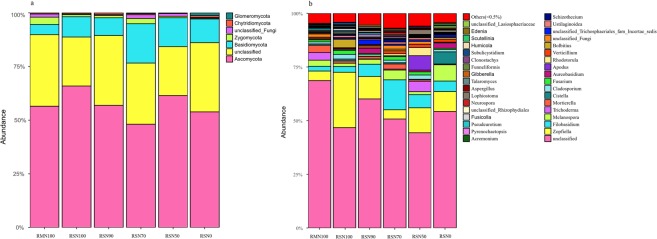
Relative abundance of phyla (**a**) and genera (**b**) in treatments expressed as percentage of the total number of rarefied ITS sequences, classified using RDP, at a confidence threshold of 50%. Genera with abundance <0.5% are grouped as “others”.

Eumycota fungi were clustered at the phylum level. The histogram of community structure, constructed according to operational taxonomic unit (OTU) sequence abundance after clustering (Fig. 2a), reflected structural and abundance differences among treatments. There were seven phyla of eumycota with abundance >0.01% in the different treatments, where the two most dominant were *Ascomycota* and *Basidiomycota*, accounting for >60% of all phyla (Fig. 2a). Abundance of *Ascomycota* was greater in the rice-fish system than the rice monoculture under an N application rate of 325.5 kg ha^−1^.

**Figure 3:**
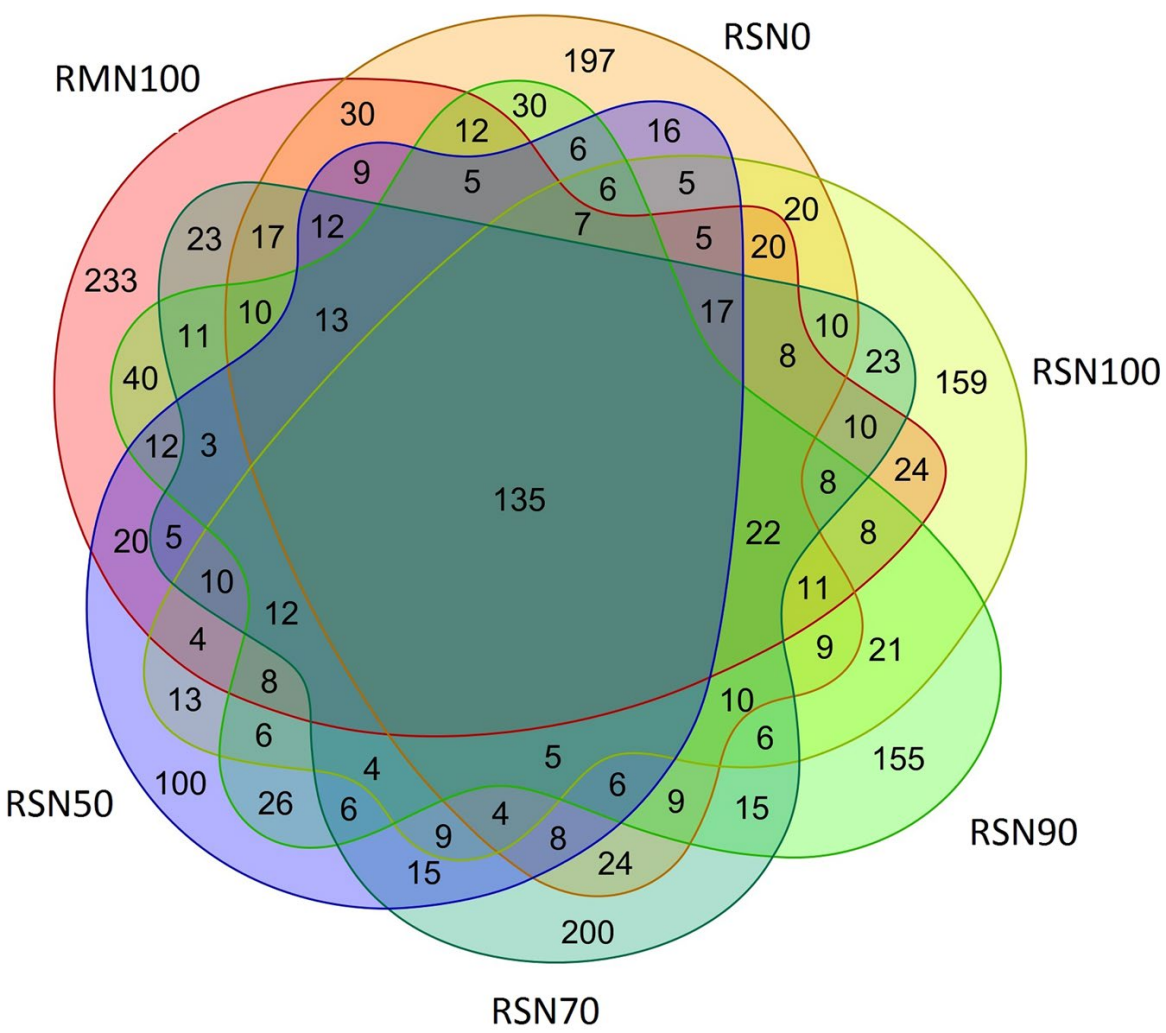
Venn diagram of OTUs in the treatments.

We found that relative abundance of *Zygomycota* in the rice-fish system increased with decreasing N when the N application rate was >227.85 kg ha^−1^, but decreased when <227.85 kg ha^−1^. However, there was no pattern in abundance of *Ascomycota*, *Basidiomycota*, *Chytridiomycota* or *Glomeromycota* among treatments. To understand within-phylum responses to N, we analysed genera in the treatments and found the most abundant, in decreasing order, were *Zopfiella*, *Filobasidium*, *Melanospora*, *Trichoderma* and *Mortierella*, where relative abundance of *Zopfiella* and *Filobasidium* was greater in the rice-fish system (Fig. 2b). A Venn diagram covering 97% of the OTU sample table data was used to visualise the similarity and overlap of the number of OTUs (N = 3900) among treatments (Fig. 3). The results showed that 3.5% of OTUs were common to all treatments, while the number of unique OTUs in RMN100 and RSN100 was 233 and 159, respectively.

### Association between soil fungi and soil physicochemical factors

The effect N application rate on soil nutrient status was investigated and we found there were differences in soil total potassium (TK) (*P* < 0.05), and salt content (Salinity) (*P* < 0.01). The lowest level of TK and highest level of salinity were observed in RSN100, and there were no treatment effects on the yield of rice or fish (Table 2).

**Table 2:** Analysis of variation (ANOVA) for the effects of treatments on soil physicochemical properties, and rice and fish biomass.

Factors	N application rate		Rice-fish mutualistic system	
	*F*	*P*	*F*	*P*
Organic matter (%)	0.461	0.763	0.205	0.674
TN (%)	0.857	0.521	0.054	0.827
TP (%)	0.136	0.965	0.305	0.61
TK (%)	4.433	0.026*	7.321	0.054
AMN (mg kg^−1^)	0.58	0.684	0.065	0.812
NiN (mg kg^−1^)	0.584	0.682	0.39	0.566
AHN (mg kg^−1^)	1.5	0.274	0.735	0.44
AP (mg kg^−1^)	1.84	0.198	6.108	0.069
AK (mg kg^−1^)	2.698	0.093	4.172	0.111
pH	0.331	0.851	0.173	0.699
Salinity (%)	13.781	0.000**	0.029	0.872
Straw fresh weight (kg ha^−1^)	0.034	0.997	2.441	0.193
Rice yield (kg ha^−1^)	0.946	0.477	6.532	0.063
Fish yield (kg ha^−1^)	2.438	0.115	—	—

Bold values indicate significant effects. *And **represent significant difference at the 0.05 and 0.01 level, respectively. TN, total nitrogen; TP, total phosphorus; TK, total potassium; AMN, ammonium nitrogen; NiN, nitrate nitrogen; AHN, alkali-hydrolysable-N; AP, available phosphorus; AK, available potassium.

**Figure 4:**
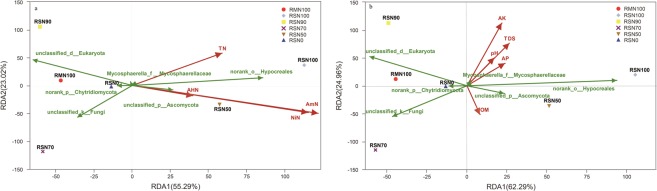
Redundancy analysis (RDA) of effect of different forms of nitrogen (**a**) and soil physicochemical properties (**b**) on soil fungi community structure. The green arrow represents fungi species, and the red arrow indicates quantitative environmental factors.

The relationship between soil physicochemical factors and fungi community structure was investigated using redundancy analysis (RDA). The different coloured dots and shapes in Fig. 4 represent different treatment sample groups; green arrows represent fungi species and red arrows represent quantitative environmental factors. Arrow length reflects the degree of influence of a given environmental factor on species data, angles between environmental factor arrows indicate positive or negative correlations, and the distance from projection origin points represent the relative influence of environmental factors on sample community distribution. Analysis of different forms of N showed the first principal axis represented 55.3% of the variation in fungi community structure (Fig. 4a), while the principal axis in the analysis of other soil chemicals explained 62.3% of the variation (Fig. 4b). Ammonium nitrogen and nitrate nitrogen (Fig. 4a), and high N application rate and available K (Fig. 4b) were the key drivers of soil fungi communities.

## Discussion

Diverse soil fungi communities are essential in the maintenance of soil ecosystem function, and are affected by tillage, land use and alien species invasion^17–19^. Rice-fish mutualistic farming systems may offer a sustainable solution to agricultural production, however, whether such systems promote or inhibit biomass and diversity of fungi in paddy soils remains poorly understood. While many studies have suggested that rice-fish production systems promote the activity of aquatic animals, leading to an increase in biomass and diversity of soil fungi^15^, there is also evidence of a lack of change or reduction in fungi biomass, diversity, and activity in paddy soils in the presence of aquatic animals, due to body surface secretions from animals^6^. Our results confirmed that cultivation of the fish *Monopterus albus* in rice fields reduced the number of decomposer fungi because it directly altered soil physicochemical properties (Table 2). Differences in cultivation systems are often associated with differences in soil fungal communities. For example, fungi diversity in paddy soils subjected to long-term water flooding was reported to result in lower fungal diversity compared with corresponding arable soils^20^. In rice-fish systems, one potential reason for the diminished fungal community could be that the presence of fish promotes mineralisation of soil organic matter and consumption of paddy soil oxygen, and thus inhibits the growth of aerobic fungi^21,22^. Our current research identified changes in fungi community structure; the abundance of *Basidiomycota* increased, and *Filobasidium* and *Zopfiella* became dominant (Fig. 2). Keszthelyi *et al*.^17^ found that the basidiomycetous yeast *Filobasidium* secreted a toxin against the pathogenic fungus *Cryptococcus neoformans* and, with the recent discovery of new species of *Filobasidium*, the potential for its application as a biological control agent has attracted attention^18^. *Zopfiella* spp. produce pyrrolidone compounds (Zopfiellamides) that elicit antibacterial activity and inhibit pathogenic bacteria, such as *Escherichia coli*, *Bacillus subtilis* and *Candida albicans*. According to previous studies, variation in fungi populations may be a response to environmental conditions, such as pH and the abundance of amino acids, micromolecular sugars and fatty acids^15,16,19,23^. However in our rice-fish mutualistic system, soil pH did not differ significantly between treatments.

In addition to the type of production system, N application rate affected fungi community structure in the paddy soil. Although there are many reports of the effects of N fertilizer on soil microbial communities in rice ecosystems, there is a lack of research on effects of interactions between N and rice-fish production on soil microbe communities. We found that although the abundance of fungi in paddy soil was lower under low N application rates (RSN90, RSN70, RSN50 and RSN0) than under the N application rate typically employed by farmers (RSN100), community diversity tended to be higher (Table 1). Rice plant growth consumes the majority of soil N, with the remainder utilized by dominant microbes with sufficient uptake capacity, and we suggest that differences in soil and climatic conditions resulted in effects of N application rate on the soil fungi that contrasted with those in previous studies. For example, there are conflicting suggestions that long-term input of inorganic N input promotes fungal reproduction^24,25^ and reduces fungal biomass^26^ in concert with changes in soil pH^27,28^. Although the relationships between N addition and characteristics of soil fungi communities have been widely studied, little is known about soil fungi community structures in rice-fish mutualistic production systems. The present study found that low levels of N application led to low soil N content (Table S2), possibly as a result of removal by rice growth and culturing of *Monopterus albus*, which might indirectly promote mineralisation of soil organic matter and consumption of a large amount of oxygen, stimulating anaerobic microorganisms, as reported in previous studies^21,22^. Rousk and Baath^29^ suggested that the C/N ratio of additions to agricultural production systems may explain differences in soil fungal and bacterial growth. Li *et al*.^30^ found that soil alkyl C content and nitrification rate explained 32% and 43% of the variation in fungal abundance, respectively. In our current research, compared with RSN100, a 30% reduction in N application (RSN 70) resulted in an increase in *Filobasidium* and a decrease in *Zopfiella* (Fig. 2b), probably because the soil C/N ratio stimulated the growth and reproduction of *Filobasidium*, but inhibited reproduction of *Zopfiella*.

In the present study, the RDA test confirmed that soil physicochemical properties in the rice-fish mutualistic production system were greater drivers of soil fungi community structure than in the rice monoculture (Fig. 4). The influence of various soil physicochemical properties on soil fungi community structure declined with a decrease in N application rate, indicating regulation of soil fungi community composition may be mediated by N application, and we found that different forms of soil N content and available P content were important regulators of *Ascomycota*, *Mycosphaerellae* and *Hypocreales*. Previous studies have shown that soil fungi community structure may be affected by a range of environmental factors, including soil physicochemical properties^15,25,27,31^, and in this study, soil content of total N, available N and P were important regulators of the dominant fungi, such as *Ascomycota*, *Mycosphaerellae* and *Hypocreales* (Fig. 4a,b). According to previous studies, soil bacteria have a strong antagonism with soil fungi in terms of competing for resources, indicating a substantial role for biotic interactions in shaping soil microbial communities^32^. However, it is important to note the proportion of unclassified fungal groups in both agricultural systems in this study was >50%, and >70% in the rice monoculture model (Fig. 2b), indicating a large number of unrecognized fungi taxa in the rice system.

In summary, rising labour costs and the demand for high yields poses many challenges for the conservation of rice-fish systems. The present study unveils some promising possibilities for reducing the application of pesticides and chemical fertilisers via ecological intensification through the use of a mutualistic system, which as benefits for both economic performance and environmental protection. Considering the strong influence of N application rate on soil fungi communities, further research on available nutrient assignment and microbial assembly in rice-fish systems is urgently required. This study also provides insight into the response of the soil fungi community to combined nutrient management and a rice-fish mutualistic approach, which may prove helpful for maintaining soil heath for sustainable agricultural productivity. As soil fungi stasis and soil resistance to microbial invasion are correlated with the level of fungal diversity^11^, exploration of the application of different groups of fungi with beneficial ecosystem functions is likely to be important for improving the sustainability of rice-fish mutualistic production systems^1,22^. For example, antagonistic fungi that control rice sheath blight and rice false smut are candidates for potential biocontrol agents that may help to achieve a reduction in chemical fertilisers and pesticides, and suppress soil-related diseases^33,34^.

## Materials and Methods

### Ethics statement

Animal studies were reviewed and approved by the Academic Committee of Shanghai Academy of Agricultural Sciences. Animal care and experimental treatments complied with the guidelines of the China Laboratory Animal Guideline for Ethical Review of Animal Welfare (GB/T35892-2018).

### Study site

All experiments were carried out at the Zhuangxing Comprehensive Experimental Station of Shanghai Academy of Agricultural Sciences, Fengxian, Shanghai, China (30°88′89′′N, 121°38′51′′E), where the average altitude was 4 m asl. Average annual precipitation, which falls principally between April and September, was 1191.5 mm; average annual temperature was 16.1 °C, with a 225-day frost-free period, and 1711.5 h of average annual sunshine. In the experimental field, a rice-fallow rotation system was adopted prior to the study. The study site was flat and the soil type was calcareous alluvium, with basic physicochemical properties of the cultivated soil (0−20 cm) as follows: organic matter 18.9 g·kg^−1^, total N 1.23 g·kg^−1^, total P 0.32 g·kg^−1^, total K 21.5 g·kg^−1^, pH 7.28, water-soluble total salt 0.25 g·kg^−1^, alkali-hydrolysale N 142 mg·kg^−1^, ammonium nitrogen 6.4 mg·kg^−1^, nitrate nitrogen 20.2 mg·kg^−1^, available P 20.2 mg·kg^−1^, available K 94.5 mg·kg^−1^, salinity 0.08%.

### Experimental design

Three replicates of six treatments in 5 m × 2 m plots were arranged in a randomised block design in an experimental plot constructed in 2015. To clarify mutualistic effects, treatments comprised a standard rice monoculture treated with 325.5 kg N ha^−1^ (RMN100) and a rice-fish mutualistic production system treated with 325.5 kg N ha^−1^, 292.95 kg N ha^−1^, 227.85 kg N ha^−1^, 162.75 kg N ha^−1^, and 0 kg N ha^−1^ (RSN100, RSN90, RSN70, RSN50 and RSN0, respectively). Standard fertilisation for rice comprised 15 t∙ha^−1^ of base commercial organic fertiliser (organic matter 413.4 g∙kg^−1^, N 17.1 g∙kg^−1^, P_2_O_5_ 12.4 g∙kg^−1^, K_2_O 12.3 g∙kg^−1^), with 450 kg ha^−1^ compound fertiliser (17-17-17), 150 kg∙ha^−1^ urea (N 46%), and topdressing applied at tillering as 75 kg∙ha^−1^ urea (N 46%), at jointing as 450 kg ha^−1^ of compound fertiliser (17-17-17), and at earing as 150 kg ha^−1^ urea (N 46%). Total nutrient input in treatments from chemical fertilisers is listed in Table 3.

**Table 3:** Nutrient input to the treatments.

Treatment	N (kg ha^−1^)	P (kg ha^−1^)	K (kg ha^−1^)
RMN100	325.5	66.8	126.4
RSN100	325.5	66.8	126.4
RSN90	292.95	66.8	126.4
RSN70	227.85	66.8	126.4
RSN50	162.75	66.8	126.4
RSN0	0	0	0

The rice-fish mutualistic system was established as shown in Fig. S1, with circular pools for fish oriented relative to the 5 m × 2 m rice plots. Nylon screen fabric was erected around the rice field along the plot to prevent *M. albus* from escaping. The screen fabric had a height of 80 cm, with 40 cm buried beneath the soil and 40 cm above the soil surface, and was fixed to pilings every 1.5 m. Fixed fish nets (40-mesh polyethylene) were installed at the infall and outfall of the rice field. We selected the disease-resistant japonica rice variety Cv. scent soft japonica and the local variety of *M. albus*, “yellow finless eels” (40 tail kg^−1^) was stocked in the pool at a density of 450 kg ha^−1^. Field management of the rice-fish mutualistic system was carried out as described for the standard rice monoculture. A typical local water regime consisting of flooding followed by midseason drainage, re-flooding, and moist irrigation was employed during the rice-growing season. Flooding was initiated 7 days before transplanting, maintained for 30 days, followed by mid-season drainage for 1 week. This was followed by re-flooding for 40 days, then maintaining moist but not waterlogged soil until 7 days before harvesting rice.

### Rice and fish yield measurements

The crop was harvested from a 4.5 m × 1.5 m net area of each plot (gross plot size 5 m × 2 m), and the surrounding 3.25 m^2^ water refuge area was discarded. Harvested grain was sun-dried thoroughly and weighed separately for each plot. The yield from each plot was then converted to kg ha^−1^. Following rice harvest, eel cages were used to catch *M. albus* individuals, stock density was adjusted to the initial density, and the average weight of total fish per plot was measured after harvesting.

### Soil sampling

After 3 years of continuous planting, soil samples were collected after rice harvest on 20 October, 2017. In each separated plot, a 2 cm diameter stainless steel soil sampler was used to collect soil samples from the 0−20 cm surface layer from 15 points along an S-shape to form a mixed composite soil sample, and then placed into a sealed, polyethylene bag in a low temperature preservation box. In the laboratory, soil samples were sieved through a 4 mm opening mesh, and large stones, rocks, gravel, plant roots and other non-soil materials were removed prior to mixing of soil samples. Soil samples were subsequently divided into two parts, of which one was air-dried prior to determination of basic physicochemical properties, and the other was stored in a freezer at −20 °C prior to high-throughput gene sequencing.

### Determination of physicochemical properties

Organic matter was measured using the potassium dichromate method, total N (TN) was measured using the Kjeldahl digestion method, total P (TP) and total K (TK) were measured with HF-HClO_4_ digestion method, and ammonium nitrogen (AMN) was determined by the KCl extraction-indigo colorimetric method. Nitrate nitrogen (NiN) was determined by the KCl extraction-ultraviolet spectrophotometry method, alkali-hydrolysable nitrogen (AHN) was measured with the alkaline hydrolysis diffusion method, available P (AP) was extracted with 0.5 mol L^−1^ NaHCO_3_ (pH 8.5) and measured with the colorimetric method. Available K (AK) was extracted with NH_4_OAc and measured with the flame photometry method, pH was determined by the electrode method in a 1:2.5 aqueous suspension, and salinity was determined by the gravimetric method^35^.

### Soil DNA extraction and fungi community analysis

Soil fungi DNA was extracted as three replicates from each sample with a FastDNA Spin Kit for Soil kit and stored at −20 °C. Fungal PCR amplification was conducted by an ABI GeneAmp 9700 instrument (Applied Biosystems, Foster City, CA, USA) using the 18 S rRNA universal primer with barcode, ITS1F-ITS1R. The corresponding primer sequence was ITS1F: 5′-CTTGGTCATTTAGAGGAAGTAA-3′ ^36^; ITS2R: 5′-GCTGCGTTCTTC ATCGATGC-3′ ^37^. The 20 μl amplification reaction system included 10 ng of template DNA, 4 μl of FastPfu Buffer (5×), 2 μl of BSA (2.5 mmol L^−1^), 0.8 μl of ITS1F (5 mol L^−1^), 0.8 μl of ITS2R (5 mol L^−1^) and 18 μl of sterile water. The amplification conditions comprised pre-denaturation for 3 min at 94 °C, followed by 30 amplification cycles of denaturation at 94 °C for 30 s, annealing for 30 s at 55 °C, extension at 72 °C for 30 s, and a final extension at 72 °C for 5 min. PCR products were detected and quantified using a blue fluorescence quantification system according to the corresponding amount of gene sequences in the sample. For each sample, PCR amplification was performed three times independently, and triplicate products were pooled in equal amounts and purified using an AxyPrep PCR Clean-up Kit (Axygen Biosciences, Union City, CA, USA). All purified PCR products were sent to Shanghai Biozeron Co., Ltd. for qPCR and Illumina Miseq sequencing.

### Real-time quantitative PCR (qPCR)

Plasmids constructed in this study harbouring an ITS1 gene fragment were extracted from *Escherichia coli* hosts using a plasmid miniprep kit (Tiangen, China). After extraction, plasmid DNA was sequenced and aligned to the NICB database, resulting in a 100% fungal lineage match (GenBank MN044804.1). Plasmid DNA concentration was measured with a Nanodrop 2000 spectrophotometer (Thermo, USA). The plasmid copy number was 8.50 × 10^7^ copies∙μl^−1^ and standard curves spanned a range from 4.25 × 10^2^ to 4.25 × 10^6^ copies∙μl^−1^. Standard reactions were performed for all samples in triplicate using an ABI 7500 sequence detection system (Applied Biosystems, Canada) using the SYBR green quantitative PCR (qPCR) method. Each qPCR mixture (25 μl) comprised 12.5 μl of Maxima SYBR green/Rox qPCR master mix (Fermentas, Lithuania), 1 μl of each primer (10 μM), 5 μl of template DNA, and ddH_2_O (5.5 μl). All reactions were performed in 8-strip thin-well PCR tubes equipped with ultraclean cap strips (ABgene, UK). The specificity of qPCR amplification was determined by melting curve and gel electrophoresis analyses. In all experiments, the same procedure was applied to negative controls without template DNA to eliminate contamination. The abundance of each gene was calculated based on the resulting standard curves and converted to copies per gram of soil, assuming a DNA extraction efficiency of 100%.

### Statistical optimisation of sequencing data and biometric analysis

Purified amplicons were pooled in equimolar amounts and subjected to paired-end sequencing on an Illumina MiSeq platform (Illumina, San Diego, USA) according to standard protocols distributed by Majorbio Bio-Pharm Technology Co. Ltd. (Shanghai, China). Trimmomatic (version 0.33) was used to quality-filter raw fastq files, and these were merged by FLASH (version 1.2.7) with the following criteria: (i) reads were truncated at all sites with an average quality score <20 over a 50 bp sliding window; (ii) Sequences with an overlap >10 bp were merged; (iii) Sequences for each sample were separated according to barcodes (exactly matching) and primers (allowing two mismatching nucleotides), and reads containing ambiguous bases were removed. Operational taxonomic units (OTUs) were clustered with a similarity cutoff of 97% using the UPARSE pipeline (version 7.1) with a novel ‘greedy’ algorithm that performs simultaneous chimera filtering and clustering of OTUs^38^. After high-throughput sequencing and optimization, we obtained 705272 sequences from the six treatments (N = 18), with 214845304 bp, where the average base length was 304.6 bp. Representative fungal OTU sequences were identified using the UNITE database^39^. Sequence data have been deposited in the National Center for Biotechnology Information (NCBI) Sequence Read Archive (SRA) under accession number SRP149298.

### Data analysis

The effects of treatments on physicochemical soil properties and fungi diversity were investigated using one-way analysis of variance (ANOVA) followed by least significance difference (LSD) tests in SPSS 17.0 (SPSS Inc., Chicago, IL, USA). QIIME (1.7.0) software was used to calculate alpha and beta diversity of fungi, and fungi OTUs were used to characterise alpha diversity^40^. PCoA of the unweighted UniFrac distance between the samples was used to characterise the similarity (beta diversity) of fungi communities among the treatments^41^. The vegan data package within R software (version 3.5.1 for Windows, Lucent Technologies, Holmdel, NJ, USA) was used for redundancy analysis (RDA) that was used to identify factors that affected fungi community structure.

## Supplementary information

Supplementary data

## References

[1] Berg H, Tam NT (2018). Decreased use of pesticides for increased yields of rice and fish-options for sustainable food production in the Mekong Delta. ScTEn.

[2] Mohanty RK, Jena SK, Thakur AK, Patil DU (2009). Impact of high-density stocking and selective harvesting on yield and water productivity of deepwater rice–fish systems. Agric. Water Manage..

[3] Feng J, Li F, Zhou X, Xu C, Fang F (2016). Nutrient removal ability and economical benefit of a rice-fish co-culture system in aquaculture pond. Ecol. Eng..

[4] Hu L (2013). The productivity of traditional rice–fish co-culture can be increased without increasing nitrogen loss to the environment. Agric. Ecosyst. Environ..

[5] Lu J, Li X (2006). Review of rice–fish-farming systems in China — One of the globally important ingenious agricultural heritage systems (GIAHS). Aquaculture.

[6] Jones RT (2009). A comprehensive survey of soil acidobacterial diversity using pyrosequencing and clone library analyses. Isme J..

[7] Alica Montesting-Navarro MV (2016). & Jose Ignacio Querejeta Soil fungi promote nitrogen transfer among plants involved in long-lasting facilitative interactions. Perspect. Plant Ecol. Evol. Syst..

[8] Satapathy BS, Singh T, Pun KB, Rautaray SK (2014). Assessment of high yielding varieties of rice (Oryza sativa) for integrated rice-fish-horticulture farming system under lowland ecosystem of north eastern. Ann. Agric. Res. New Series.

[9] Ahmed N, Bunting SW, Rahman S, Garforth CJ (2014). Community-based climate change adaptation strategies for integrated prawn-fish-rice farming in Bangladesh to promote social-ecological resilience. Reviews in Aquaculture.

[10] Anyusheva M, Lamers M, La N, Nguyen VV, Streck T (2012). Fate of pesticides in combined paddy rice-fish pond farming systems in northern Vietnam. J Environ Qual.

[11] Oehme MF, Razzak MA, Dewan S, Becker K (2007). Studies on nitrogen cycling under different nitrogen inputs in integrated rice-fish culture in Bangladesh. Nutr Cycl Agroecosyst.

[12] Datta A, Nayak DR, Sinhababu DP, Adhya TK (2009). Methane and nitrous oxide emissions from an integrated rainfed rice–fish farming system of Eastern India. Agric. Ecosyst. Environ..

[13] Hu Z (2016). A comparison of methane emissions following rice paddies conversion to crab-fish farming wetlands in southeast China. Environ. Sci. Pollut. Res. Int..

[14] Bonanomi G, Capodilupo M, Incerti G, Gaglione SA, Scala F (2014). Fungal diversity increases soil fungistasis and resistance to microbial invasion by a non-resident species. Biol. Control.

[15] Nayak PK (2018). Ecological mechanism and diversity in rice based integrated farming system. Ecol. Indicators.

[16] Wang M (2017). Influence of peanut cultivars and environmental conditions on the diversity and community composition of pod rot soil fungi in China. Mycobiology.

[17] Keszthelyi A, Hamari Z, Pfeiffer I, Vágvölgyi C, Kucsera J (2008). Comparison of killer toxin-producing and non-producing strains of *Filobasidium capsuligenum*- proposal for two varieties. Microbiol. Res..

[18] Merín, G., Moratade, L. M. & Morata, V. I. Pectinolytic yeasts from viticultural and enological environments: Novel finding of *Filobasidium capsuligenum* producing pectinases. *J. Basic Microbiol*. **8** (2014). 10.1002/jobm.201200534 23686851

[19] Bärlocher F, Boddy L (2016). Aquatic Fungal Ecology- How does it differ from terrestrial. Fungal Ecol..

[20] Chen L (2016). The influence of soil properties on the size and structure of bacterial and fungal communities along a paddy soil chronosequence. Eur. J. Soil. Biol..

[21] Mirhaj M, Razzak MA, Wahab MA (2014). Comparison of nitrogen balances and efficiencies in rice cum prawn vs. rice cum fish cultures in Mymensingh, North-Eastern Bangladesh. Agr. Syst..

[22] Xie J (2011). Ecological mechanisms underlying the sustainability of the agricultural heritage rice-fish coculture system [sustainability science]. PNAS.

[23] Chen J (2018). Environmentally friendly fertilizers: A review of materials used and their effects on the environment. ScTEn.

[24] Wang JC, Song Y, Ma TF, Raza W (2017). Impacts of inorganic and organic fertilization treatments on bacterial and fungal communities in a paddy soil. Appl. Soil Ecol..

[25] Geisseler D, Scow KM (2014). Long-term effects of mineral fertilizers on soil microorganisms – A review. Soil Biol. Biochem..

[26] Chen J (2013). Biochar soil amendment increased bacterial but decreased fungal gene abundance with shifts in community structure in a slightly acid rice paddy from Southwest China. Appl. Soil Ecol..

[27] Kirchmann H, Schön M, Börjesson G, Hamnér K, Kätterer T (2013). Properties of soils in the Swedish long-term fertility experiments: VII. Changes in topsoil and upper subsoil at Örja and Fors after 50 years of nitrogen fertilization and manure application. Acta Agriculturæ Scandinavica.

[28] Jiang Y (2016). Crop rotations alter bacterial and fungal diversity in paddy soils across East Asia. Soil Biol. Biochem..

[29] Rousk J, Bååth E (2007). Fungal and bacterial growth in soil with plant materials of different C/N ratios. FEMS Microbiol. Ecol..

[30] Li YC (2017). Bamboo invasion of broadleaf forests altered soil fungal community closely linked to changes in soil organic C chemical composition and mineral N production. Plant Soil.

[31] Linquist BA, Adviento-Borbe MA, Pittelkow CM, van Kessel C, van Groenigen KJ (2012). Fertilizer management practices and greenhouse gas emissions from rice systems: A quantitative review and analysis. Field Crops Res..

[32] Mohammad B (2018). Structure and function of the global topsoil microbiome. Nature.

[33] Bass D, Richards TA (2011). Three reasons to re-evaluate fungal diversity ‘on Earth and in the ocean’. Fungal Biol. Rev..

[34] Bruggen AH, Semenov AM (2000). In search of biological indicators for soil health and disease suppression. Appl. Soil Ecol..

[35] Lu, R. K. Soil argrochemistry analysis protocoes. China Agriculture Science Press: Beijing (1999).

[36] Gardes M, Bruns TD (2010). ITS primers with enhanced specificity for basidiomycetes–application to the identification of mycorrhizae and rusts. Mol. Ecol. Resour..

[37] Baldwin BG (1992). Phylogenetic utility of the internal transcribed spacers of nuclear ribosomal DNA in plants: an example from the compositae. Mol. Phylogenet. Evol..

[38] Edgar RC (2013). UPARSE: highly accurate OUT sequences from microbial amplicon reads. Nat. Methods.

[39] Abarenkov K (2010). The UNITE database for molecular identification of fungi-recent updates and future perspectives. New phytol..

[40] Schloss PD (2009). Introducing mothur: Open-Source, Platform-Independent, Community-Supported Software for Describing and Comparing Microbial Communities. Appl. Environ. Microbiol..

[41] Anderson MJ, Trevor JW (2003). Canonical analysis of principal coordinates: A useful method of constrained ordination for ecology. Ecology.

