# Supplementary material for: Effect of nitrogen application rate on soil fungi community structure in a rice-fish mutualistic system
Source: Sci Rep. 2019 Nov 7;9:16188. doi: 10.1038/s41598-019-52602-x (PMC6838126; doi:10.1038/s41598-019-52602-x)
Supplement: Supplementary file 1 — Supplementary data [file 41598_2019_52602_MOESM1_ESM.pdf]

Supplementary data

Supplementary data for the article "Effect of nitrogen application rate on soil fungi community structure in a rice-fish mutualistic system" by Shumei Cai[1], Weiguang Lv[1], Haitao Zhu, Deshan Zhang, Zishi Fu, Hanlin zhang & Sixin Xu[*]

Corresponding Author:

Sixin Xu, Institute of Eco-Environment and Plant Protection, Shanghai Academy of Agricultural Sciences, 1000 Jinqi Road, Shanghai 201403, P. R. China. Phone: +86-21-62202441; Fax: +86-21-62201112; Email: xsxofsaas@163.com.

A TABLE OF CONTENTS

Fig. S-1 Schematic diagram of the rice-fish system.

Table S-1 Means comparison on soil physicochemical variables as affected by cultivation systems.

Table S-2 Means comparison on soil physicochemical variables as affected by N application rate.

Table S-3 P values of the RDA within selected N factors.

Table S-4 P values of the RDA within selected other environmental factors

Table S-5 The construct of standard plasmid in qPCR

Table S-6 18S rRNA gene copy numbers by qPCR

Fig. S-2 qPCR standard curve of fungi 18S rRNA genes

Fig. S-3 qPCR amplification curve of fungi 18S rRNA genes

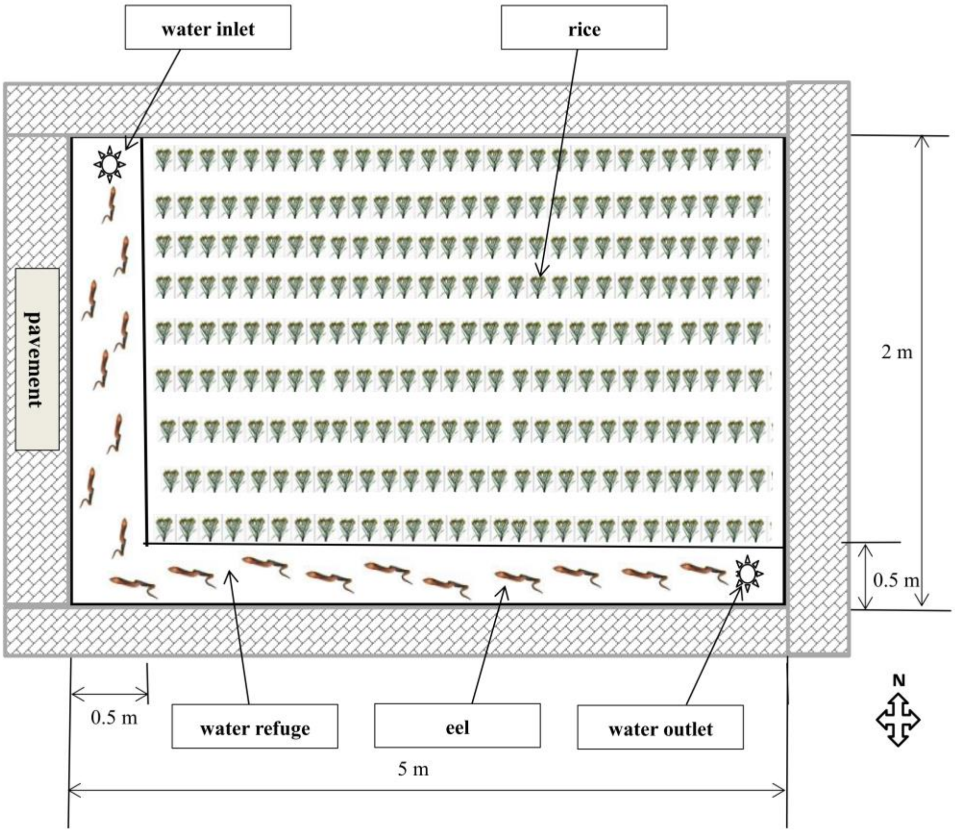

Fig. S-1 Schematic diagram of the rice-fish system

Table S-1

Means comparison on soil physicochemical variables as affected by cultivation systems

Treatment	Rice monoculture (RMN100)	Rice-fish symbiotic (RSN100)
TK (%)	2.14±0.06ns	1.96±0.02
Salinity (%)	0.19±0.02ns	0.19±0.01
organic matter (%)	1.70±0.26ns	1.57±0.11
TN (%)	0.13±0.02ns	0.12±0.01
TP (%)	0.03±0.00ns	0.03±0.00
AMN (mg kg^{-1})	20.46±2.54ns	21.88±4.94
NiN (mg kg^{-1})	6.70±0.93ns	7.76±1.41
AHN (mg kg^{-1})	85.92±6.17ns	80.28±2.30
AP (mg kg^{-1})	3.20±0.13ns	2.25±0.36
AK (mg kg^{-1})	146.67±17.64ns	183.33±3.33
pH	6.97±0.17ns	7.04±0.04
straw fresh weight (kg ha^{-1})	8906.78±1024.04ns	6989.68±675.83
Rice yield (kg ha^{-1})	7114.36±185.92ns	6097.64±351.71

Value (means±standard deviation, n=3) within a row followed by ns indicates no significant difference between the cultivation treatments.

Table S-2

Means comparison on soil physicochemical variables as affected by N application rate

Treatment	RSN100	RSN90	RSN70	RSN50	RSN0
TK (%)	1.96±0.02b	2.09±0.03ab	2.09±0.06ab	2.25±0.07a	2.12±0.06ab
Salinity (%)	0.19±0.01a	0.15±0.02a	0.09±0.00b	0.09±0.01b	0.17±0.01a
organic matter (%)	1.57±0.11a	1.57±0.23a	1.63±0.13a	1.79±0.08a	1.56±0.12a
TN (%)	0.12±0.01a	0.11±0.01a	0.10±0.00a	0.12±0.01a	0.11±0.01a
TP (%)	0.03±0.00a	0.03±0.00a	0.03±0.00a	0.03±0.00a	0.03±0.00a
AMN (mg kg^{-1})	21.88±4.94a	16.58±0.94a	19.06±2.12a	21.87±3.53a	20.46±0.71a
NiN (mg kg^{-1})	7.76±1.41a	6.35±0.61a	7.06±0.35a	7.41±0.61a	7.76±0.35a
AHN (mg kg^{-1})	80.28±2.3a	74.63±4.93a	76.21±7.33a	87.33±7.5a	90.16±3.86a
AP (mg kg^{-1})	2.25±0.36a	1.98±0.36a	1.30±0.24a	2.52±0.47a	2.52±0.41a
AK (mg kg^{-1})	183.33±3.33a	203.33±23.33a	143.33±13.33a	146.67±17.64a	170.00±11.55a
pH	7.04±0.04a	7.05±0.04a	7.02±0.09a	6.98±0.06a	6.98±0.04a
straw fresh weight (kg ha^{-1})	6989.68±675.83a	7121.69±351.92a	7262.03±939.31a	7306.37±638.26a	7142.02±678.09a
Rice yield (kg ha^{-1})	6097.64±351.71a	6369.99±58.55a	6219.64±370.7a	6573.33±284.52a	6854.01±357.72a
Fish yield (kg ha^{-1})	2770.39±76.25a	2795.01±49.94a	2752.96±43.67a	2657.57±24.89a	2599.11±57.77a

Value (means±standard deviation, n=3) within a row followed by different lowercase indicates difference at $P < 0.05$ between N application treatments.

Table S-3 P values of the RDA within selected N factors

Factors	RDA1	RDA2	r^2	P
**TN**	0.707	0.707	0.24	0.667
**AmN**	0.896	-0.444	0.74	0.125
**NiN**	0.892	-0.451	0.67	0.183
**AHN**	0.878	-0.479	0.08	0.886

Table S-4 P values of the RDA within selected other environmental factors

Factors	RDA1	RDA2	r^2	P
**OM**	0.157	-0.988	0.15	0.789
**AP**	0.509	0.861	0.12	0.853
**AK**	0.191	0.982	0.73	0.122
**pH**	0.324	0.946	0.158	0.792
**TDS**	0.328	0.9447	0.367	0.494

Table S-5 The construct of standard plasmid in qPCR

**Plasmid type**	**Plasmid name**	**DNA length (bp)**	**plasmid Concentration ($ng·\mu l^{-1}$)**	**40-fold diluent DNA Concentration ($ng·\mu l^{-1}$)**	**Calculated equation**	**Copy number ($copies·\mu l^{-1}$)**
ITS	pMD18-T	2974	11.09	0.277	Copy number ($copies·\mu l^{-1}$) = [DNA concentration($ng·\mu l^{-1}$) $\times 10^{-9} \times 6.02 \times 10^{23}$] / (DNA length×660)	8.50×10^{7}

Table S-6 18S rRNA gene copy numbers by qPCR

No.	Treatments	Average quantity (10^3 copies·μl^{-1})	DNA volume (μl)	Soil extraction weight (g)	Gene copy number (10^6 Copies·g^{-1} soil)
**1**	RMN100	6.56	50	0.287	1.14
**2**	RMN100	6.02	50	0.28	1.08
**3**	RMN100	12.60	50	0.272	2.32
**4**	RSN100	3.1 4	50	0.276	0.57
**5**	RSN100	5.5	50	0.286	0.96
**6**	RSN100	11.5	50	0.289	1.99
**7**	RSN90	3.37	50	0.27	0.62
**8**	RSN90	2.86	50	0.282	0.51
**9**	RSN90	3.95	50	0.278	0.71
**10**	RSN70	7.1	50	0.28	1.27
**11**	RSN70	3.08	50	0.281	0.55
**12**	RSN70	6.06	50	0.282	1.07
**13**	RSN50	2.44	50	0.285	0.43
**14**	RSN50	4.55	50	0.281	0.81
**15**	RSN50	2.57	50	0.27	0.48
**16**	RSN0	7.32	50	0.28	1.31
**17**	RSN0	1.75	50	0.269	0.33
**18**	RSN0	4.09	50	0.276	0.74

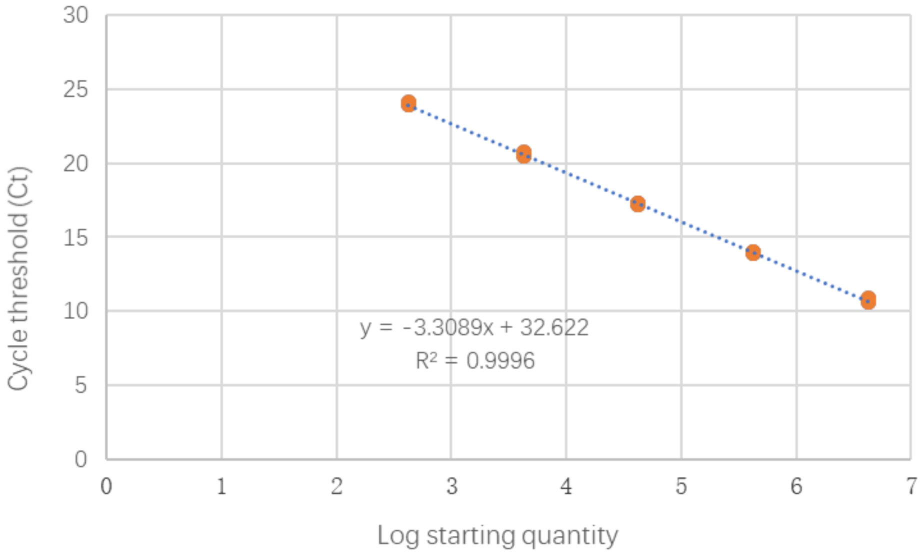

Fig. S-2 qPCR standard curve of fungi 18S rRNA genes

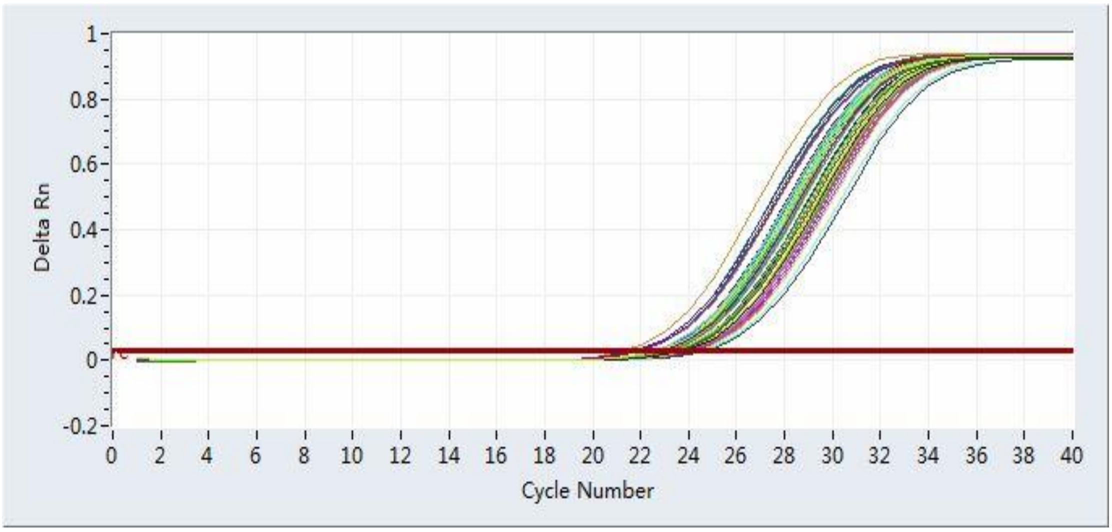

Fig. S-3 qPCR amplification curve of fungi 18S rRNA genes
